# New Insight into the Anti-liver Fibrosis Effect of Multitargeted Tyrosine Kinase Inhibitors: From Molecular Target to Clinical Trials

**DOI:** 10.3389/fphar.2015.00300

**Published:** 2016-01-18

**Authors:** Kai Qu, Zichao Huang, Ting Lin, Sinan Liu, Hulin Chang, Zhaoyong Yan, Hongxin Zhang, Chang Liu

**Affiliations:** ^1^Department of Hepatobiliary Surgery, The First Affiliated Hospital of Medical College, Xi’an Jiaotong UniversityXi’an, China; ^2^Department of General Surgery, Shaanxi Cancer HospitalXi’an, China; ^3^Department of Hepatobiliary Surgery, Shaanxi Provincial People’s HospitalXi’an, China; ^4^Department of Pain Treatment, Tangdu Hospital, Fourth Military Medical UniversityXi’an, China

**Keywords:** tyrosine kinase inhibitors, liver fibrosis, clinical trials, preclinical study, molecular mechanisms

## Abstract

Tyrosine kinases (TKs) is a family of tyrosine protein kinases with important functions in the regulation of a broad variety of physiological cell processes. Overactivity of TK disturbs cellular homeostasis and has been linked to the development of certain diseases, including various fibrotic diseases. In regard to liver fibrosis, several TKs, such as vascular endothelial growth factor receptor, platelet-derived growth factor receptor, fibroblast growth factor receptor, and epidermal growth factor receptor kinases, have been identified as central mediators in collagen production and potential targets for anti-liver fibrosis therapies. Given the essential role of TKs during liver fibrogenesis, multitargeted inhibitors of aberrant TK activity, including sorafenib, erlotinib, imatinib, sunitinib, nilotinib, brivanib and vatalanib, have been shown to have potential for treating liver fibrosis. Beneficial effects are observed by researchers of this field using these multitargeted TK inhibitors in preclinical animal models and in patients with liver fibrosis. The present review will briefly summarize the anti-liver fibrosis effects of multitargeted TK inhibitors and molecular mechanisms.

## Introduction

Liver fibrosis is a chronic medical condition in which the normal liver architecture is replaced by fibrous tissue, scar and regenerative nodules leading to loss of liver function due to various etiologies including infection, drug, cholestasis, metabolic disorder, or immune attack (Hernandez-Gea and Friedman, 2011). Liver fibrosis affects 100s of millions of patients worldwide, which ultimately resulting in cirrhosis, hepatocellular carcinoma (HCC), or even death. Although liver fibrosis is generally recognized being potentially reversible and a number of therapies have been investigated in animal models, those diverse anti-fibrotic therapies are not seemingly effective from bench to bedside. Till date, treatment of liver fibrosis depends upon the stage of the disease, and liver transplantation is the only curative therapy for end stage of liver cirrhosis (Adam and Hoti, 2009). A thorough understanding of the underlying mechanism is critical for developing effective therapeutic approach for cirrhotic patients.

## Tyrosine Kinases Involved in Liver Fibrogenesis

Grateful thanks to the decades of relevant experiments and researches, a numerous molecules and signaling pathways involved in the liver fibrogenesis were unveiled and corresponding therapeutics were taken root (Friedman, 2008). Among them, a family of proteins called tyrosine kinases (TKs) are found to be involved in this process. TKs can be divided into two subgroups, receptor tyrosine kinases (RTKs) and non-receptor tyrosine kinases (nRTKs). RTKs include vascular endothelial growth factor receptor (VEGFR), platelet-derived growth factor receptor (PDGFR), fibroblast growth factor receptor (FGFR), and epidermal growth factor receptor (EGFR) kinases. Meanwhile, nRTKs include c-Abl and Src kinases. Both RTKs and nRTKs are found to be essential for cellular signal transduction networks (Xu and Huang, 2010).

The RTKs are membrane receptors that activate intracellular signaling pathways upon ligand binding to their extracellular domains. These receptors are single-transmembrane proteins comprising an extracellular ligand-binding domain and a linked cytoplasmically oriented, catalytic domain (Almendro et al., 2010). The activation process of RTKs is triggered by the dimerisation of two RTK monomers as well as autophosphorylation of the intracellular phosphatase domain to increase the catalytic activity, which consequently generates a biochemical message and activates intracellular signaling pathways.

**FIGURE 1:**
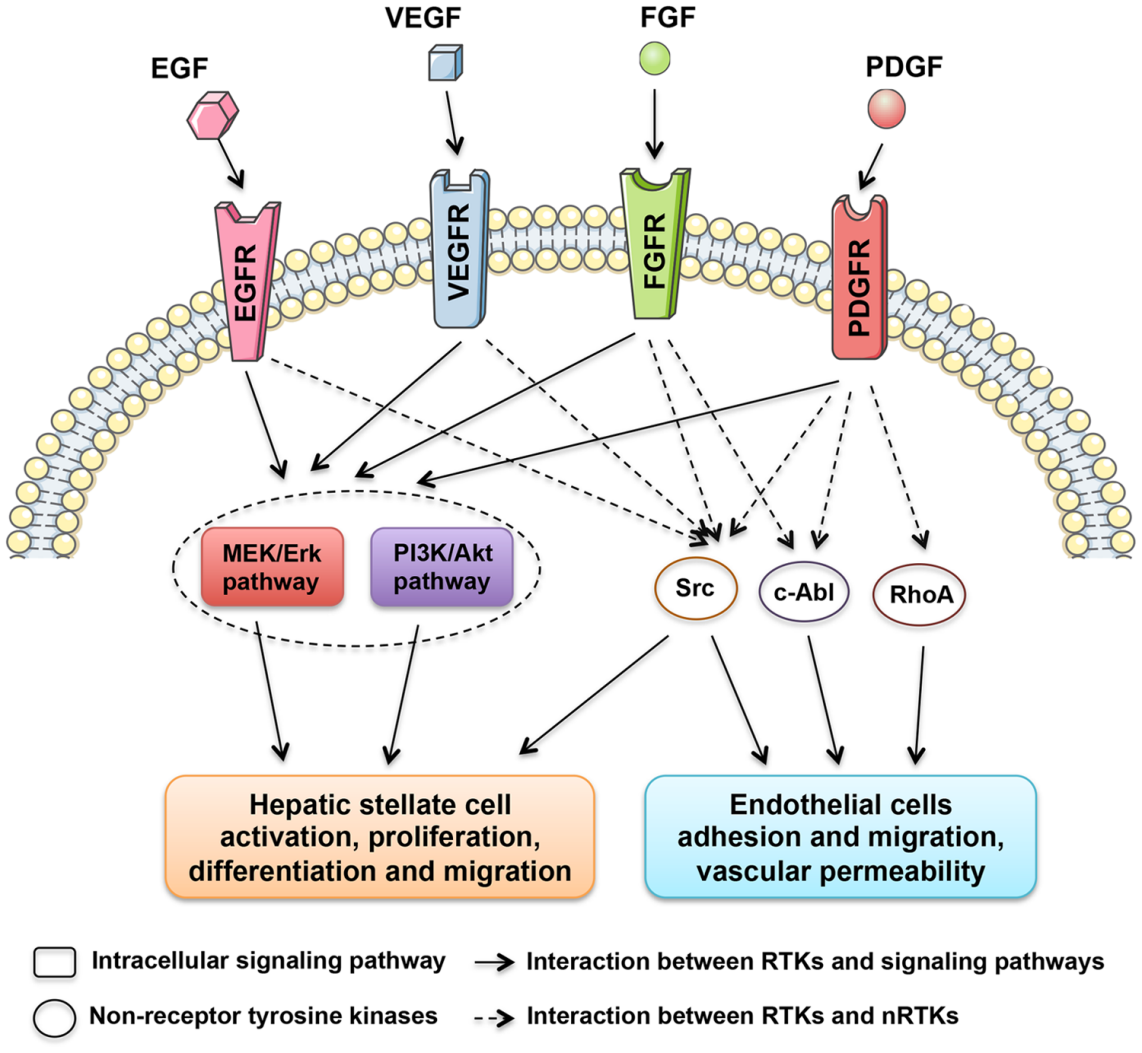
**Tyrosine kinases with central roles in liver fibrosis.** The regulatory network is composed of receptor tyrosine kinases, including EGFR, VEGFR, FGFR, and PDGFR kinases, and non-receptor tyrosine kinases, such as Src, c-Abl, and RhoA kinases, which stimulate the activation of HSCs and intrahepatic angiogenesis.

In contrast to RTKs, nRTKs (Src, c-Abl, and RhoA) lack extracellular and transmembrane domains, and only include a catalytic domain and a regulatory domain (Arora and Scholar, 2005; Zander and Hallek, 2011). nRTKs modulate signaling pathways after activation in the cytoplasm use different regulatory mechanisms. Additionally, it is also found that nRTKs can be activated by RTKs (**Figure 1**). The interaction between RTKs and nRTKs therefore contribute together to modulate cellular differentiation and proliferation.

### Tyrosine Kinases as Modulators of Hepatic Stellate Cells Activation

It’s widely accepted that a hallmark of liver fibrogenesis is the transdifferentiation of resting hepatic stellate cells (HSCs) into a myofibroblastic cell type. It was found that many TKs were expressed in activated HSCs. Moreover, the expression of several TKs, especially PDGFR (Heldin, 2014), VEGFR (Yoshiji et al., 2003), and EGFR (Fuchs et al., 2014), were significantly increased during the course of liver fibrosis development. Because of the critical roles in key signal transduction, TKs therefore harbor a mitogenic potential, which when activated, result in the transformation of resting HSCs to active HSCs. As shown in **Figure 1**, multiple downstream signaling pathways, such as MEK/ERK and PI3K/Akt pathways, are found to be activated by TKs during HSC activation. Many TK targeting agents exhibit significant inhibitory effects on chemotaxis, activation and collagen synthesis pathways in HSCs.

### Tyrosine Kinases as Modulators of Intrahepatic Angiogenesis

Alterations in the hepatic vasculature are also defined as a crucial component during liver fibrogenesis. Established evidence clearly indicates that microvascular abnormalities promotes portal hypertension and liver fibrosis progression (Medina et al., 2004; Thabut and Shah, 2010). In parallel with capillarization of hepatic sinusoids, intrahepatic angiogenesis giving rise to shunts between pre- and post- sinusoidal vessels would lead to increased portal vascular resistance and decreased effective hepatocyte perfusion (Yoshiji et al., 2003). To date, many TKs have been identified joining in angiogenesis during liver fibrosis progression. Among these, VEGFRs are the most potent in the angiogenesis process (**Figure 1**). VEGFR expression significantly increased during the course of liver fibrosis development in experimental studies (Yan et al., 2015). Anti-VEGFR treatment using either antibodies (Yoshiji et al., 2003) or agents (Yang et al., 2014) significantly attenuates liver fibrosis progression. Additionally, PDGFRs were also considered as proangiogenic molecules involved in portal hypertension and might be potential targets for anti-fibrotic therapy (Rosmorduc, 2010).

## Anti-Fibrotic Activity of Multitargeted Tyrosine Kinase Inhibitors

Over the past decade, numerous small molecule inhibitors targeting TKs have been developed (**Table 1**). Initially, these synthesized drugs were developed for anti-tumor therapy. In recent years, the application of multi-targeted TK inhibitors has also dramatically changed the conventional treatment modes for many other non-malignant diseases, especially for fibrotic diseases (Beyer and Distler, 2013; Heldin, 2014). Given the central role of TKs in liver fibrosis, blockade of the TKs appears to be a promising anti-fibrotic treatment approach. Currently, significant benefits of multitargeted TK inhibitors in liver fibrosis have been observed in preclinical experiments on animal models (Rossler et al., 2008; Grimminger et al., 2010). In the following part, we will summarize recent findings of anti-liver fibrosis effects of TK inhibitors (**Table 2**).

**Table 1:** IC50 values for TK inhibitors inhibition *in vitro*.

TK inhibitors	IC50 values for TK inhibitors inhibition *in vitro* (nM)									
	
	VEGFR-1	VEGFR-2	VEGFR-3	PDGFR-α	PDGFR-β	FGFR-1	EGFR	c-Kit	Fit-3	Bcr/Abl
Sorafenib	NR	15	20	NR	57	580	>10000	68	58	NR
Erlotinib	NR	NR	NR	NR	NR	NR	2	NR	NR	NR
Imatinib	>10000	>10000	>10000	100	100	NR	NR	100	>10000	600
Sunitinib	NR	80	NR	NR	2	>1000	>1000	NR	NR	NR
Nilotinib	NR	NR	NR	NR	NR	NR	NR	NR	NR	<30
Brivanib	380	25	NR	NR	>1000	148	>1000	NR	NR	NR
Vatalanib	77	37	660	NR	580	NR	NR	730	NR	NR


*TK inhibitors, tyrosine kinase inhibitors; NR, not reported. Data taken from http://www.selleckchem.com/.*

**Table 2:** Summary of anti-liver fibrosis effects of TK inhibitors in preclinical studies.

TK inhibitors	Effects on fibrotic processes								Animal models of liver fibrosis used for evaluation
		
	HSC activation	Angiogenesis	CCl_4_	BDL	TAA	DEN	DMN	NASH	PCLS	Pig serum	Parasite
Sorafenib	**√**	**√**	**√**	**√**	**√**	**√**	**√**	**√**	**√**		
Erlotinib	**√**		**√**	**√**		**√**					
Imatinib	**√**		**√**	**√**	**√**				**√**	**√**	**√**
Sunitinib	**√**	**√**	**√**						**√**		
Nilotinib	**√**		**√**	**√**	**√**						
Brivanib	**√**	**√**	**√**	**√**	**√**			**√**			
Vatalanib	**√**		**√**								


*CCl_4_, carbon tetrachloride; BDL, bile duct ligation; DEN, diethylnitrosamine; DMN, dimethylnitrosamine; TAA, thioacetamide; NASH, non-alcoholic steatohepatitis model; PCLS, precision-cut liver slices from fibrotic livers; Parasite, Schistosoma mansoni.*

### TK Inhibitors in Clinical Trials as Anti-liver Fibrosis Agents

#### Sorafenib

As one of the most intensively investigated multitargeted TK inhibitors, Sorafenib mainly targets Raf/ERK, VEGFR, and PDGFR-β pathways. Mechanistic investigation demonstrated that sorafenib exhibited potential anti-cancer activities by inhibiting cellular proliferation, suppressing angiogenesis and inducing apoptosis in various tumor types (Plastaras et al., 2007). Clinical trails further revealed that sorafenib can be used alone as the first treatment for advanced HCC. Interestingly, during the course of anti-HCC treatment, clinicians observed positive side effects of sorafenib on liver cirrhosis (Mejias et al., 2009). The anti-fibrotic effect of sorafenib is clearly demonstrated by numerous experiment studies. In nearly all animal models of liver fibrosis, such as carbon tetrachloride (CCl_4_), bile duct ligation (BDL), dimethylnitrosamine (DMN), diethylnitrosamine (DEN), or thioacetamide (TAA) induced models, sorafenib exhibits anti-liver fibrosis effects (Hennenberg et al., 2009; Wang et al., 2010; Thabut et al., 2011; Hong et al., 2013; Westra et al., 2014a; Liu et al., 2015; Stefano et al., 2015; **Table 2**).

Hepatic stellate cells are recognized as the main matrix-producing cells and being responsible for excessive deposition of extracellular matrix components during liver fibrogenesis. Mechanistic investigations revealed that sorafenib inhibited PDGF-BB-induced cellular proliferation in a dose-dependent manner in HSCs (Wang et al., 2010). The anti-proliferation of sorafenib on HSCs are found to be mediated by downregulating expression of cyclins and cyclin dependent kinases (CDKs) and inhibiting the phosphorylation of ERK and Akt (Plastaras et al., 2007; Tomizawa et al., 2010). Recently, increasing evidence have shown that enhanced intrahepatic angiogenesis is associated with faster fibrosis progression and thus has been identified as a crucial contributor to the fibrogenesis. Thabut et al. (2011) also reported that sorafenib was capable of inhibiting the Kruppel-like factor (KLF6)-Angiopoietin-1 (Ang1)-fibronectin molecular triad, thereby suppressing intrahepatic angiogenesis and attenuating liver fibrosis (Thabut et al., 2011).

In preclinical experiments, sorafenib is also found to attenuate the complications of liver cirrhosis. Portal hypertension is a life-threatening complication of liver disease defined by a portal venous pressure gradient exceeding 5 mm (Lee and Kim, 2007). Preclinical studies showed that sorafenib treatment resulted in a reduction in portal pressure and angiogenesis in BDL rats without affecting systemic blood pressure (Tugues et al., 2007; Mejias et al., 2009; Rosmorduc, 2010). Hennenberg et al. (2009) found that the effect of sorafenib on intrahepatic angiogenesis and portal hypertension is mediated by Rho kinase activity (Hennenberg et al., 2009). Additionally, it is also be observed that sorafenib may influence hepatopulmonary (Chang et al., 2013; Yang et al., 2014) and hepatic encephalopathy syndrome (Hsu et al., 2012) in cirrhotic rats.

In early clinical trials of sorafenib as anti-HCC agent, it was observed that patients with liver cirrhosis who received sorafenib therapy had a decrease in portal venous flow of at least 36% (Coriat et al., 2011). Similarly, in a small clinical study, Pinter et al. (2012) also reported the protective effect of a 2-weeks sorafenib treatment on portal hypertension in HCC patients with liver cirrhosis (Pinter et al., 2012). Additionally, Theysohn et al. (2012) found that sorafenib reduced hepatopulmonary shunt in patients with liver cirrhosis, which might greatly improve the prognosis of these patients (Theysohn et al., 2012). Recently, a multi-center, placebo-controlled randomized clinical trial of the effect of sorafenib on portal pressure in patients with cirrhosis was carried out (NCT01714609, **Table 3**). Researchers recruited patients with cirrhosis who have high portal vein pressure and treated them using sorafenib (400 mg twice daily) or placebo. Results from this clinical trial might supply evidence for clinicians to use sorafenib as anti-liver fibrosis agent.

**Table 3:** Tyrosine kinase inhibitors in clinical trials as anti-liver fibrosis agents.

TK inhibitors	ClinicalTrials.gov identifier	Recruited parcitipants	Intervention	Study phase	Status
Sorafenib	NCT01714609	Liver cirrhosis participants with portal hypertension	Sorafenib 400mg p.o. twice daily	Phase II	Completed
Erlotinib	NCT02273362	Liver cirrhosis participants following HCC resection	Erlotinib p.o. for 7 days	Phase I	Recruiting


#### Erlotinib

Erlotinib was the second EGFR TK inhibitor approved by the FDA for non-small cell lung cancer (NSCLC). Fuchs et al. (2014) observed that erlotinib, used at doses equivalent to or less than those used in humans, significantly reduced fibrogenesis in three different animal models of progressive cirrhosis: DEN or BDL induced rat model and CCl_4_ induced mouse model. They also found that erlotinib reduced the number of activated HSCs by depressing EGFR phosphorylation in HSCs. An undergoing clinical trial (NCT02273362, **Table 3**) is conducted to evaluate the effects of erlotinib in fibrogenesis inhibition and HCC prevention.

### Other TK Inhibitors Exhibited Potential Anti-liver Fibrosis Activity in Preclinical Experiments

#### Imatinib

Imatinib (also known as STI571), is a potent, competitive 2-phenylamonioyrimideine class inhibitor of three TKs, PDGFR, Bcr-Abl, and c-Kit. It is initially developed for the treatment of chronic myeloid leucemia (CML) and gastrointestinal stroma tumors (by targeting c-Kit). Given its inhibitory capacity on PDGFR which plays an critical role in the activation of fibroblasts, imatinib therefore is considered as a potential therapeutic candidate for the treatment of fibrotic diseases. Akhmetshina et al. (2009) found that imatinib did not only prevent but also reverse established fibrosis in systemic sclerosis models. Apart from SSc, the anti-fibrotic effects of imatinib were consequently observed in pulmonary, renal and liver fibrosis (Daniels et al., 2004; Abdollahi et al., 2005; Wang et al., 2005; Yoshiji et al., 2005). In many animal models of liver fibrosis, such as CCl_4_, BDL, TAA, or Schistosoma mansoni induced liver fibrosis, imatinib exhibits anti-liver fibrosis effects (Yoshiji et al., 2005; Neef et al., 2006; El-Agamy et al., 2011; Shaker et al., 2011a; Kuo et al., 2012; Shiha et al., 2014). In a pig serum-induced liver fibrosis model, Yoshiji et al. (2005) found that imatinib attenuated liver fibrosis via suppressing HSCs activation. In addition, imatinib exhibits increased anti-liver fibrosis activities when used in combination with an angiotensin-converting enzyme inhibitor (ACE-I), perindopril, which suppresses TGF-β1 expression (Yoshiji et al., 2006). Westra et al. (2014a,b) conducted an *in vitro* model using prolonged culture of precision-cut liver slices to screen antifibrotic drugs. It was also found that Imatinib could significantly decrease the expression of fibrosis markers, such as α-SMA, Pcol1A1, and Hsp47 (Westra et al., 2014b).

It should be noted that, different from sorafenib, imatinib seems to only reduce early liver fibrogenesis but does not prevent progression in the long term. In a study reported by Neef et al. (2006), it was found that prophylactic imatinib markedly reduced fibrosis in the first 3 weeks after BDL. Early imatinib treatment induced a 50% decrease of MMP-2 activity and TIMP-1 expression in HSCs, but left numbers of activated HSCs unchanged (Neef et al., 2006). Moreover, when imatinib was used in advanced fibrosis models, it neither reduced numbers of activated HSCs nor inhibit extracellular matrix production.

#### Sunitinib

Sunitinib is an oral indolin-2-one structural analog, which inhibits multiple RTKs such as VEGFR1/2/3, PDGFR-α/β, FGFR, and c-Kit (Faivre et al., 2007). Clinical trials revealed that sunitinib had potent anti-tumor and anti-angiogenesis effects in multiple cancer types. In liver fibrosis models, sunitinib has been shown to decrease inflammatory infiltration and expression of fibrosis markers in fibrotic livers (Tugues et al., 2007; Westra et al., 2014a). A *in vitro* study conducted by Majumder et al. (2013) revealed that sunitinib inhibited collagen synthesis in HSCs by 47%, attenuated HSC contraction by 65%, and reduced cell migration by 28%. In addition, they also found that sunitinib suppressed angiogenic capacity of endothelial cells (ECs). Similarly, it was also observed that sunitinib could decrease the number of vascular cell adhesion molecule-1 (VCAM-1) and intercellular adhesion molecule-1 (ICAM-1) positive staining hepatic vessels, and consequently reduced portal vein pressure in cirrhotic rats (Tugues et al., 2007).

#### Nilotinib

Nilotinib, a selective BCR-ABL TK inhibitor, is shown to be 30-fold more potent than imatinib in preclinical *in vitro* studies. Shaker et al. (2011a,b,c, 2013) found that nilotinib had a promising anti-fibrotic activity in experimental models of liver fibrosis by inhibiting activation of HSCs (Shiha et al., 2014). Liu et al. (2011) also reported that nilotinib significantly inhibited PDGF and TGF-β-simulated activation of ERK and Akt and consequently reduced collagen deposition and α-SMA expression in CCl_4_ and BDL-induced fibrotic models.

#### Brivanib

Brivanib is an orally available dual inhibitor of VEGF and FGF signaling. Nakamura et al. (2014) evaluated the anti-liver fibrosis effects of brivanib on three experimental fibrotic mouse models, including BDL, CCl_4_, and chronic TAA induced mouse models of fibrosis. Lin et al. (2014) further found that brivanib markedly suppressed intrahepatic angiogenesis and portal hypertension in cirrhotic rats. Similarly, Yang et al. (2014) also observed brivanib improved hepatic blood flow and inhibited ascites formation in NASH-cirrhotic rats.

#### Vatalanib

Vatalanib (also known as PTK787/ZK22258) is found to mainly target VEGFR-1 and VEGFR-2, and it also inhibits the activity of PDGFR-β, Flt-4, c-kit, and c-fms with less potency. In liver fibrosis models, Liu et al. (2009a,b) reported that vatalanib attenuated stellate cell activation and liver fibrosis progression by inhibiting VEGF signaling as well as targeting of the PDGF and TGF-β-signaling pathways.

## Hepatotoxicity of TK Inhibitors: An Important Issue Limited Their Clinical Use

Most of TK inhibitors are metabolized in liver by hepatic cytochrome P450 enzyme system (Druker, 2003; Lathia et al., 2006; Peer et al., 2012), implying a potential hepatotoxicity when they are administrated in patients. Iacovelli et al. (2014) conducted a meta-analysis base on 3691 patients who received TK inhibitors treatment and found hepatotoxicity occurred in 23–40% of patients treated with TK inhibitors. It is been found that hepatotoxicity usually occurred within the first 2 months after TK inhibitors treatment (Shah et al., 2013). Fatality from TK inhibitor-induced hepatotoxicity is less common compared to hepatotoxic drugs in other classes, but may lead to unfavorable events including liver cirrhosis and even liver failure (Cross et al., 2006; Schramm et al., 2008; Tonyali et al., 2010; Shah et al., 2013). In the following aspect, we summarized the hepatotoxicity of TK inhibitors that observed in clinical cases.

Sorafenib is reported to exhibit a high degree of inter-individual variability in pharmacokinetics and clinical efficacy. The magnitude of variability on sorafenib exposure (area under the plasma concentration-time curve, AUC) ranged from 5 to 83%, and the peak plasma concentrations varied from 33 to 88% at oral doses of 200 or 400 mg administrated twice daily. The median time to peak plasma concentration varied from 2 to 9.5 h (Awada et al., 2005; Clark et al., 2005; Moore et al., 2005; Strumberg et al., 2007). Hepatotoxicity was reported during the therapy periods in some clinical cases. Llanos et al. (2009) reported a case of sorafenib-induced severe hepatotoxicity in a 73-years-old man with Child-Pugh A hepatitis-C virus-related cirrhosis and multinodular HCC. Schramm et al. (2008) also reported a case of sorafenib-induced liver failure. In addition, acute liver failure caused by imatinib and sunitinib have also been observed (Cross et al., 2006; Tonyali et al., 2010; Shah et al., 2013).

Due to the activity of TKs plays an essential role in many physiological processes and its inhibition by TK inhibitors may lead to side effects as discussed above. Therefore, targeting liver fibrosis via specific delivery of TK inhibitors to HSCs might reduce side effects. Gonzalo et al. (2007) conducted a HSC-selective carrier mannose-6-phosphate modified human serum albumin (M6PHSA) to combine with a TK inhibitor which exhibited potent anti-fibrotic effects. Their findings supply a promising approach to attenuate liver fibrogenesis using TK inhibitors.

## Perspective

Many intracellular signaling pathways are activated inappropriate during fibrogenesis, in which the activation of TKs is recognized the initial trigger for HSC activation and intrahepatic angiogenesis. The treatment for liver fibrosis, in the past, tends to focus on only one target. As a result, poor benefits obtained despite non-corresponding efforts. Nowadays, accumulating preclinical experiments of multitargeted TK inhibitors made it possible to analyze and look forward to whether TK inhibitors have beneficial effects on not only malignant tumors but also fibrotic disease. Clinical trials of two TK inbhitors (sorafenib and erlotinib) have been carried out and encouraging results have already obtained. Based on the advantages of multitargeted TK inhibitors, targeted therapy might become major approaches for treating liver fibrosis in future.

It also should point out that the usefulness of TK inhibitors for long term treatment of liver fibrosis depends on the severity of the side effects. Although the most common adverse effects of TK inhibitors including rash, gastrointestinal symptoms, fatigue, edema, and neurological symptoms are generally mild and tolerable for liver fibrosis patients, liver function impairment and even acute liver failure have been observed in some clinical cases. Specific delivery of TK inhibitors to selective cells, such as HSCs, might be promising approach to attenuate liver fibrosis in future. Besides, the high price of TK inhibitors might also limit their application on liver fibrosis.

Given together, TK inhibitors are efficient not only on malignant tumors, but also on some non-malignant diseases, especially liver fibrosis. In the near future, clinical application of TK inhibitors on liver fibrosis will turn out to be not merely an efficient but also safety treatment.

## Author Contributions

All authors fulfill the authorship requirements and have approved the final version of the anuscript. KQ, ZH, and CL developed the paper design and revised the manuscript; SL and HC collected samples and performed literature search; ZY and HZ participated in research work and analyzed data; KQ, ZH, and TL wrote the first draft of the manuscript to which all authors made significant subsequent contributions.

## Conflict of Interest Statement

The authors declare that the research was conducted in the absence of any commercial or financial relationships that could be construed as a potential conflict of interest.
